# Fast and Non-Destructive Quail Egg Freshness Assessment Using a Thermal Camera and Deep Learning-Based Air Cell Detection Algorithms for the Revalidation of the Expiration Date of Eggs

**DOI:** 10.3390/s22207703

**Published:** 2022-10-11

**Authors:** Victor Massaki Nakaguchi, Tofael Ahamed

**Affiliations:** 1 Graduate School of Science and Technology, University of Tsukuba, Tennodai 1-1-1, Tsukuba 305-8577, Ibaraki, Japan; 2 Faculty of Life and Environmental Sciences, University of Tsukuba, Tennodai 1-1-1, Tsukuba 305-8577, Ibaraki, Japan

**Keywords:** quail eggs, thermal camera, poultry production, fresh eggs, quality assessment, YOLOv5, deep learning

## Abstract

Freshness is one of the most important parameters for assessing the quality of avian eggs. Available techniques to estimate the degradation of albumen and enlargement of the air cell are either destructive or not suitable for high-throughput applications. The aim of this research was to introduce a new approach to evaluate the air cell of quail eggs for freshness assessment as a fast, noninvasive, and nondestructive method. A new methodology was proposed by using a thermal microcamera and deep learning object detection algorithms. To evaluate the new method, we stored 174 quail eggs and collected thermal images 30, 50, and 60 days after the labeled expiration date. These data, 522 in total, were expanded to 3610 by image augmentation techniques and then split into training and validation samples to produce models of the deep learning algorithms, referred to as “You Only Look Once” version 4 and 5 (YOLOv4 and YOLOv5) and EfficientDet. We tested the models in a new dataset composed of 60 eggs that were kept for 15 days after the labeled expiration label date. The validation of our methodology was performed by measuring the air cell area highlighted in the thermal images at the pixel level; thus, we compared the difference in the weight of eggs between the first day of storage and after 10 days under accelerated aging conditions. The statistical significance showed that the two variables (air cell and weight) were negatively correlated (R^2^ = 0.676). The deep learning models could predict freshness with F1 scores of 0.69, 0.89, and 0.86 for the YOLOv4, YOLOv5, and EfficientDet models, respectively. The new methodology for freshness assessment demonstrated that the best model reclassified 48.33% of our testing dataset. Therefore, those expired eggs could have their expiration date extended for another 2 weeks from the original label date.

## 1. Introduction

The agricultural supply chain has been passing through a digital transformation over the last few years by absorbing elements from industry 4.0 [1]. Innovations linked to sensor technology, telecommunications, robotics, the Internet of Things (IoT), and artificial intelligence (AI) are being applied to management automation and real-time data-driven intervention. In addition, these revolutions toward enhancing the production of food, fibers, and energy are expected to provide solutions for the contention of wastes and the mitigation of environmental pollutants [2]. Novel practices for agri-food industries introducing computational methods combined with smart devices are allowing new alternatives to assess the quality of farming products and traceability. Hence, with the recent release of 5th generation telecommunications networks (5G), for the very first time, rural areas can obtain access to fast internet connections, which may support farmers and decision-makers in the adoption of best practices and mediation just in time.

In the field of computer sciences, AI is a cutting-edge technology from recent decades with the potential to disrupt society in the coming years. Deep neural networks (DNNs) are the workhorse of AI that have been leading solutions to nonlinear and multidimensional problems, such as image processing, natural language processing, and speech recognition. In addition, a combination of big data, faster algorithms, and powerful processing units are considered to be mainly responsible for bringing the deep learning (DL) approach to the spotlight, and DL is currently considered the state of the art in human-centered AI systems.

In the meantime, the greatest efforts have been focused on computer vision to address image classification, object detection, segmentation, and localization. Faster algorithms have been improved continuously to reach high confidence and speed by employing convolutional neural networks (CNNs) on the backbone of those algorithms. Currently, the state of the art object detection algorithms are based on the You Only Look Once (YOLO) [3]. A series of improvements on this deep learning algorithm have achieved fast inference on edge devices, including smartphones, low-end computers, and cloud processing platforms [4]. YOLO is classified as a one-stage detector algorithm [5], the same class as RetinaNet [6] and SSD [7]; however, the architecture is anchor-based and inherited from two-stage detectors, such as the R-CNN family [8], Fast R-CNN [9], and Faster R-CNN [10].

YOLO was introduced in 2015 [3]; the breakthrough of this object detection algorithm was its ability to predict classes and localize coordinates in images (bounding boxes) by using a single CNN, which makes the inference faster and real-time applications become possible. The basic idea behind YOLO architecture was to look at the entire image (or frame) all at once, then divide it into a grid S × S, after which, the localization of an object is treated as a regression problem instead of a traditional classification problem. When the center of an object falls into a specific grid, that grid becomes responsible for detecting the object [11].

Since its release, YOLO has evolved into a series, also known as versions. The first 3 versions of YOLO were released by Redmon et al. [3,12,13], and the 4th version (YOLOv4) by Bochkovskiy et al. (2020) [5]; this last one was reported to overperform the previous version (YOLOv3) by 12% in speed and 10% for accuracy, and then became one of the most used real-time solution object detection algorithms.

Over time, the upgraded versions have stepped-up the tradeoff between speed and accuracy of detections, and besides that, from the version 5 (referred to as YOLOv5), the YOLO algorithms have incorporated user-friendly characteristics, including less complex framework, training efficiency, and portability of models among diverse inference platforms [14]. Computer vision systems based on the YOLO series have been reported for solutions related to detecting fruits in orchards using YOLOv3 [15], YOLOv4 [16], and YOLOv5 [17], leaf diseases detections [18], defects assessment on fruits [19], and pest detection [20].

Furthermore, for other agricultural purposes, computer vision systems based on deep learning object detection algorithms show enormous potential, which includes self-driving vehicles [21,22], robotics [23], and object tracking [24,25]. However, most of the utilization of vision systems has been concerned with in-farm operations aiming to supply labor shortages and quality of operations; few implementations are given to reduce waste at the shelf level as a priority target.

Egg quality is a complex task to be assessed because it involves diverse parameters, such as size, weight, color, eggshell defects, spoilage, bacterial infection, and freshness (which can be considered the major criteria for quality [26]).

The freshness domain is explained as an objective attribute that represents biochemical and physical variables that are sensory [27]. The lack of fresh characteristics occurs principally due to aging effects that start to compromise egg quality immediately after oviposition. The loss of water and CO_2_ through shell pores reduces the weight of eggs [28] and increases the pH [29], respectively, liquefying the albumen and yolk, and therefore facilitating osmotic exchange between them [30]. These factors can be intensified by environmental conditions, especially temperature and humidity [31], during the storage period.

Several methods to evaluate the freshness of eggs have been reported, including destructive analysis, such as the Haugh unit (HU) [32], the pH of the albumen [33], the yolk index (YI) [34], and the air cell size [35]. Nondestructive techniques mainly involve NIR/Raman spectroscopy [30,36,37], odor sensors [38,39,40], electrical conductivity [41], ultrasound [42], and candling [43]. However, most of these techniques are not robust for real-time applications, and the nondestructive analysis techniques are not even suitable due to issues regarding equipment cost, intraclass variations related to shell color (specific calibration is required) and thickness, and environmental parameters, such as illumination, temperature, and humidity [44]. A recent study used a pulse phase thermography approach with neural networks and was able to estimate the aging of hen eggs according to the approximation of the air cell size and obtained a high degree correlation (R^2^ > 0.95) [45], which demonstrates the potential of high-throughput application of thermal imaging and heuristic algorithms.

All over the world, different countries have adopted different metrics for freshness standards. The European Union (EU), for instance, adopted air cell size as a parameter to evaluate the freshness of avian eggs [46], while in Brazil, the standard is HU [47]. Therefore, the aim of this work was to develop a fast and accurate method using a computer vision model based on deep learning algorithms for air cell detection as a fast and nondestructive method to classify nonfresh quail eggs using a thermal microcamera. Our hypothesis was that the loss in egg weight, as determined by the air cell increase, can be detected by thermal cameras due to gas transmission through the eggshell; thus, a machine vision system could be carried out by detecting this feature in a quick and nondestructive way. In this study, we dealt with deep learning object detection algorithms to assess the shelf-life quality of quail eggs toward freshness by classification of radiometric images from a thermal camera according to the new proposed methodology.

## 2. Materials and Methods

### 2.1. Experimental Environment

For this study, we used Japanese quail eggs (*Coturnix japonica*) collected from local grocery stores. The label expiration date was considered the reference for the end point of freshness. The experiments were conducted in the Bioproduction and Machinery laboratory, University of Tsukuba, Japan, during the middle of the summer season, in which the average range daily temperature was 24–32 °C. The methodology was developed in two phases: first, air cell assessment by pixel measurement; second, deep learning object detection for automatic classification of nonfresh eggs using thermal images.

### 2.2. Thermal Imaging

A FLIR^®^ (Teledyne FLIR LLC, Wilsonville, OR, USA) Model VUE™ 336, 6.8 mm, thermal camera with a sensor resolution of 336 × 256 pixels and a spectral band range of 7.5–13.5 μm, size 2.26” (5.74 cm) × 1.75” (4.44 cm), was used to collect radiometric images from the quail eggs. Thermal cameras can produce images by interpreting the intensity of infrared (IR) radiance emitted from the target when interacting with the environment. Therefore, the images result from atmospheric transmission, IR reflection, and the emission wavelength from the target [48]. In this regard, three variables are determinant to acquire information of objects: the size, distance of targets and the angle from the camera.

The main point when using thermal cameras relies on the fact of no-light dependency compared to optical cameras. Thermal cameras are specifically used for night vision problems and body temperature measurements. However, limitations are found due to low resolution and high cost compared to conventional cameras. In addition, thermal cameras provide relative temperatures, and absolute measurements can be reached after data processing or calibration procedures for specific purposes.

**Figure 1:**
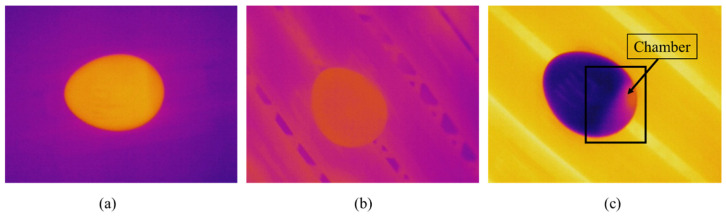
(**a**) Thermal image of an egg at room temperature. (**b**) Warmed egg at 37 °C. (**c**) Thermal picture from a cold egg at 17 °C while room temperature was 28 °C.

In this study, we used a thermal microcamera to collect the egg images. By random exploration of the thermal camera, we found that radiometric images collected from cold eggs could highlight a “chamber” on the large base of the eggs, as we knew that the air cell is located in the same position. We investigated the possibility of assessing the freshness according to the size of this feature, which could be a reference to the air cell in proportion to the aging effects on eggs (Figure 1). When the eggs are colder than room temperature, the chamber is highlighted (Figure 1c).

**Figure 2:**
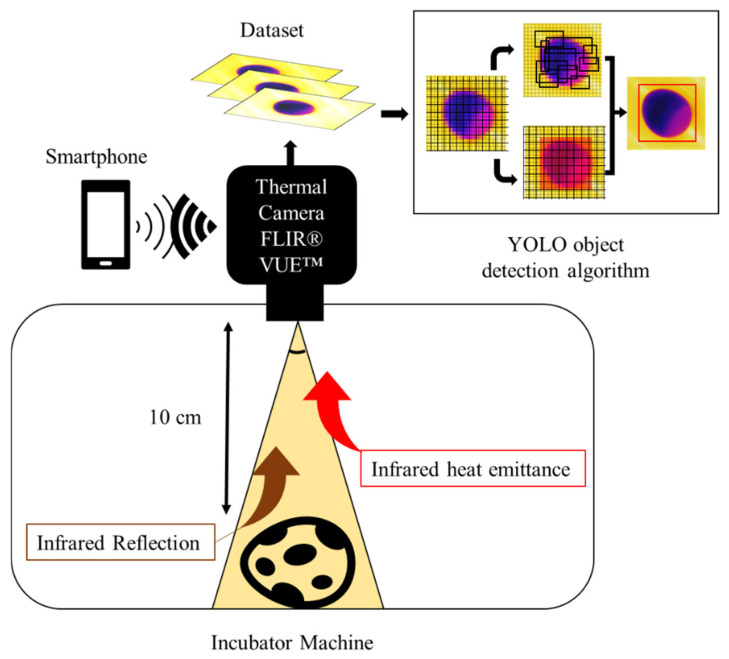
A schematic representation of the methodology proposed to collect thermal images from quail eggs.

We collected images inside an automatic incubator machine (no brand) to avoid direct atmospheric interference on the eggs. The incubator was warmed to 38 °C to enhance the temperature contrast between the environment and the cold eggs and was rewarmed after every 20 egg images. The room temperature was constant at 27 °C during the data collection period. We kept the eggs inside the incubator by picking them up by their equator region. The thermal camera was placed above the target in an up view position ± 10 cm from the eggs (Figure 2). The thermal camera was controlled with a SHARP^®^ smartphone (Sharp Corporation, Sakai, Osaka, Japan), AQUOS™ sense4 basic Model A003SH with an ANDROID™ version 11 operating system connected to the camera by Bluetooth technology. The software used was FLIR^®^ UAS™ 2 version 2.2.4.

### 2.3. Dataset Collection

We collected 390 quail eggs from local grocery stores at random. However, 8 eggs were found to be cracked; therefore, the remaining 382 eggs were used for the experiments. The eggs were divided into three groups:

The 1st group was composed of 174 eggs stored for 60 days continuously inside a conventional refrigerator under a minimum cold temperature of 17 °C and a relative humidity of 45%. On the 30th, 50th, and 60th days, the eggs were removed from the refrigerator for image sampling (thermal pictures acquired). After the 30th day of storage, we assumed that no eggs would be fresh at all; to make sure, data were collected on the 50th day and on the 60th day. In addition, by collecting samples at 3 time points, we could obtain more representative data over long storage periods. This dataset was used to train the vision-based object detection algorithms.

The 2nd group was formed of 148 eggs, and this group was used to assess the air cell size. Air cells increase due to aging effects, and there is a loss in egg weight due to that; therefore, these measures could be correlated.

The 3rd group had 60 eggs that were used for testing the prediction model. This group was stored under same conditions of group 1 and evaluated 15 days after the expiration date. To assess the air cells in this group, we boiled the eggs and visually confirmed the air cell size by cutting the eggs longitudinally.

### 2.4. Air Cell Assessment Methodology

To correlate the air cell enlargement with the loss in egg weight, an experiment was performed with eggs from group 2. Basically, we scaled fresh eggs immediately after purchase (1st day) and after a 10-day storage period under accelerated aging conditions (the eggs were kept at room temperature in summer conditions, where the average temperature of the room was 27 °C and humidity of 60%, such conditions can speed up the dehydration of eggs).

**Figure 3:**
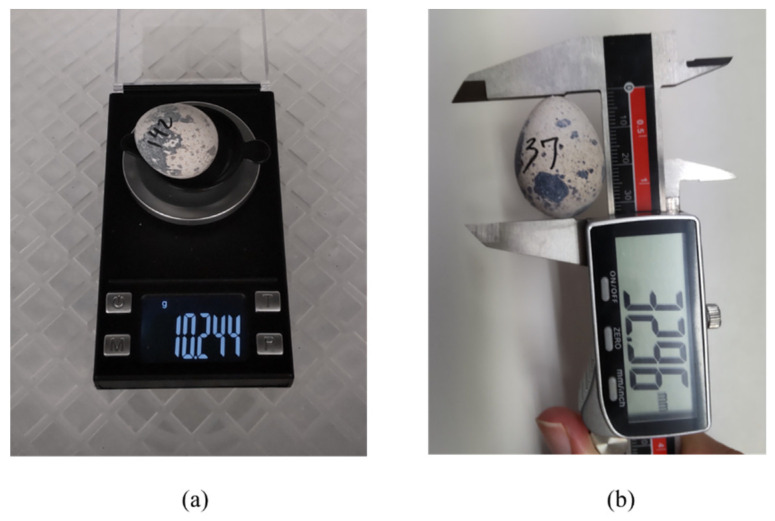
(**a**) Digital semiprecise scale. (**b**) Digital caliper measurement of the longitudinal axis.

The 148 eggs from group 2 were all numbered and scaled with a digital semiprecise scale (0.001 g precision, no brand). In addition, the long axis (Y) size was measured with a digital caliper (0.01 mm precision). Figure 3 shows the procedures.

**Figure 4:**
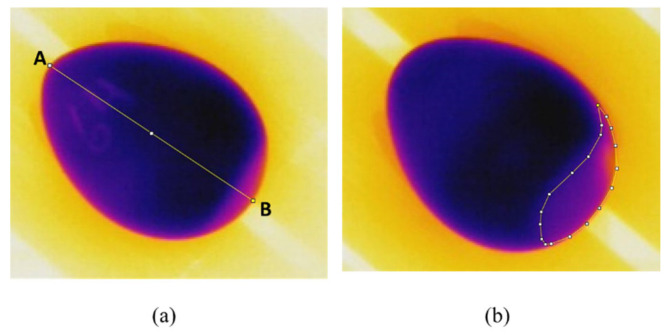
(**a**) The calibration process was performed by scaling the known distance from point “A” to point “B”. (**b**) The area was determined by calculating the number of pixels under the delimited contour in relation to the known distance defined in (**a**).

The measurements of the representative air cell on the pictures were performed manually by contouring the feature highlighted on the large base of the eggs. We used the open-source software ImageJ (64 bits, version 1.8.0) developed by Wayne Rasband and contributors from the National Institutes of Health in the United States [49]. The software could provide a conversion between real measurements and the length of pixels, according to a known real distance. Figure 4 shows the workflow procedure. First, the real distance was converted into a pixel length. Next, according to the scale (pixel/mm), the contoured area was calculated by the software.

We determined the pixel distance between two points by means of 3 line distances (three points A-B) to reduce the error and the subjectivity of pixel length conversion.

The Pearson correlation (Equation (1)) was adopted to calculate the relationship between weight and air cell size variation during the accelerated aging period.

(1) $$r=\frac{\sum \left(x_{i}-\bar{x}\right)\left(y_{i}-\bar{y}\right)\;}{\sqrt{\sum {\left(x_{i}-\bar{x}\right)}^{2}\sum {\left(y_{i}-\bar{y}\right)}^{2}}}$$

where r is the coefficient of correlation, x_i_ and y_i_ are the x and y variable samples (area and weight), respectively, and $\bar{x}$ and $\bar{y}$ are the mean values of the x-y sample variables.

### 2.5. Deep Learning-Based Object Detection Algorithms

In the field of machine learning (ML) techniques, deep learning (DL) uses deep neural networks to deal with nonlinear problems involving big data to create predictive models. In recent years, compared to traditional ML, such as logistic regression, support vector machine, and other methods, DL has been faster and more accurate when performing under multidimensional data [50], for instance, image classification, segmentation, and localization.

The complexity of DL algorithms makes us think of it as a combination of “black boxes” where the entire process is difficult to visualize in a simple way. However, YOLO is a DL object detection algorithm that uses a single convolutional neural network (CNN) to localize the object of interest inside the image and classifies the object as a regression problem.

In our problems, the training dataset was fed into YOLO (v4 and v5). The algorithm then took a look at every image at once and then divided each image into a 13 × 13 grid. As our input size was 416 × 416, each cell of the grid had 32 × 32 pixels. Thus, when a high probability of the center point of the eggs with a large air cell was located, that grid was addressed to the prediction of the “not-fresh” class. The YOLO algorithm simplified architecture workflow can be seen in Figure 2.

Since its release, YOLO has achieved many series, including YOLOv5 [51] and YOLOv6 [52], which were released by companies. Nevertheless, peer-reviewed research articles have not yet been published. However, regardless of that, the community of developers and industry are providing solutions adopting these tools. YOLOv5 was used in this study due to its stability and portability (deployment capability), considering the immediate potential of our methodology that can be extended to mobile applications addressing the most common deployment formats, such as TensorFlow™ Lite and Edge TPU. The first 4 versions of YOLO [5] were based on the Darknet framework, and the 5th version uses the PyTorch framework, which is based on a Python ecosystem, one of the most used programing languages worldwide [53], especially in the data science field.

A few elements were modified from YOLOv4 to YOLOv5, including the modified bottom-up and top-down layers in the new feature pyramid network (FPN) [54] inside the path aggregation network (PANet) [55] on the neck of the algorithm. Another modification was the loss function; the 5th version uses the binary cross entropy with the logit loss function [56].

**Figure 5:**
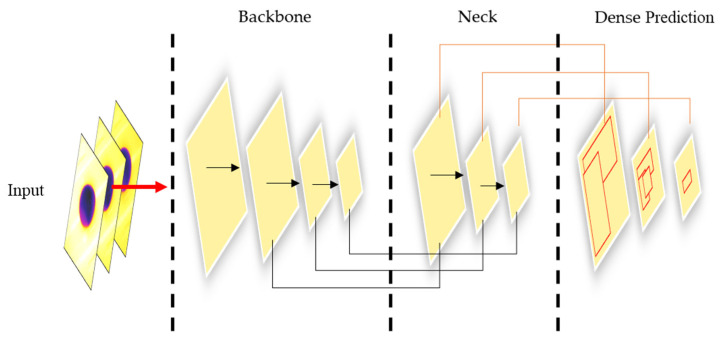
One-stage detectors YOLOv4, YOLOv5, and EfficientDet basic architecture for the thermal imagery-based datasets.

In this work, we trained YOLOv4, YOLOv5, and EfficientDet object detection architectures to predict nonfresh eggs after the expiration date and to revalidate the label date of the remaining eggs. The overall architecture of the deep learning algorithms is shown in Figure 5, where we compared the different structures of the object detection algorithms used in this work.

The backbone of the algorithm represents the CNN type, which was responsible for feature extraction (edges, shapes, color differences) and the creation of the feature map by using convolutional operations. The neck was a feature aggregator network; it collected those features from the backbone and put them together as bottom-up and top-down features to the head, which was the final step to predict the nonfresh egg position on the image or frame. This last part was responsible for plotting the bounding boxes around the class and labeling the image with its name. Table 1 shows a comparison between the object detection models and its basic architecture employed in this study.

EfficientDet was released by Google Research, LLC [57]. The main point of this algorithm is the light model, high accuracy, and multiscalability, which focus on efficiency when detecting small objects and the speed of detections aiming at low-end devices. EfficientDet uses the EfficientNet convolutional neural network on the backbone to extract the features related to the egg shape, color, and borders of air cells with maximum efficiency in terms of computation costs. The bidirectional feature pyramid network (BiFPN) on the neck part is an aggregator similar to PANnet for feature fusion except for some skipped connections between the pyramid network from the backbone, which also contributes to increasing the detection efficiency of our thermal features related to eggs. Finally, the box prediction network on the head is responsible for labeling the predicted class.

#### 2.5.1. Data Labeling

The first group (174 eggs) was used to train the YOLO and EfficientDet algorithms, and the total data were 522 (from the 30th, 50th, and 60th days). We considered that after 30 days of storage, all eggs would not be fresh at all. Therefore, the model of nonfresh eggs could be well-represented according to this dataset. The 522 images were enlarged by augmentation techniques to extend the generalization and to better extract features during training. We adopted spatial, pixel, and cutmix augmentation techniques.

**Figure 6:**
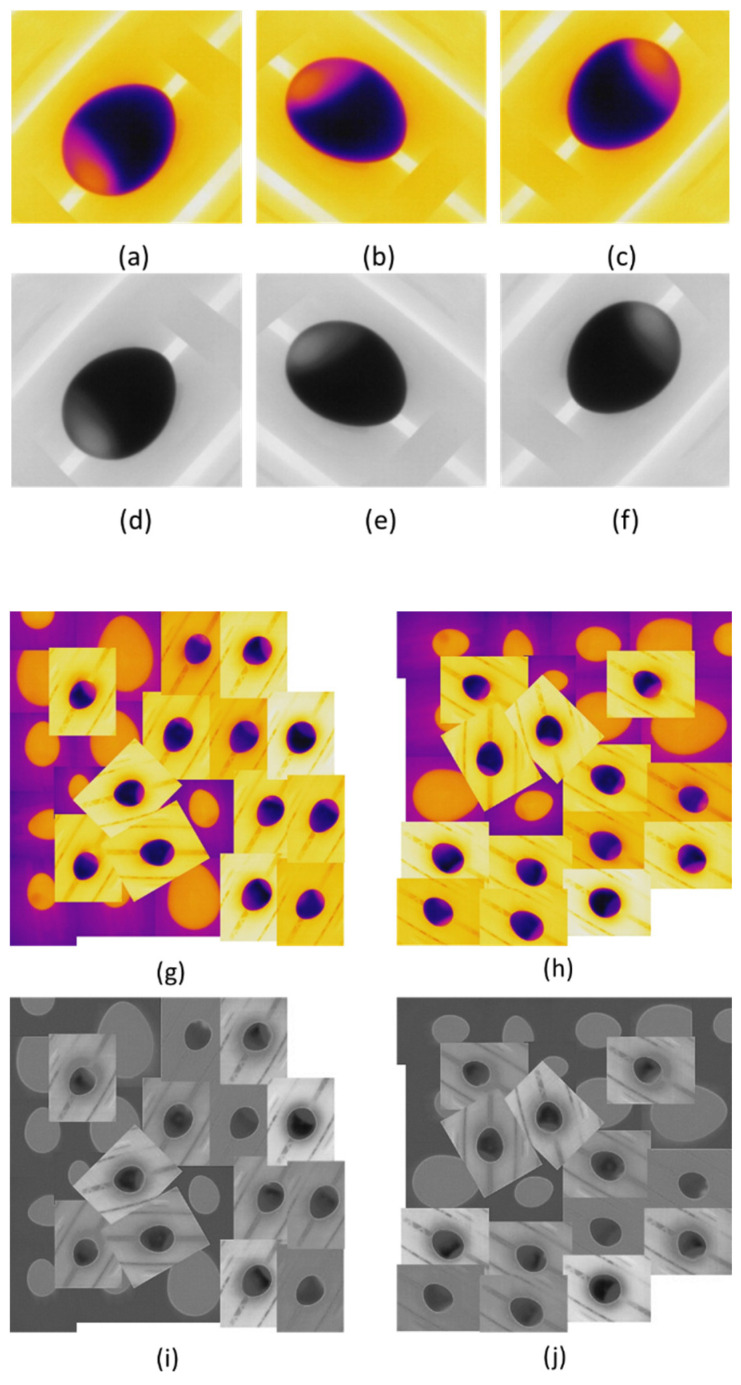
(**a**) Original image. (**b**,**c**) Free rotations applied to the original image. (**d**–**f**) Monochrome transformation for pixel augmentation. (**g**) Cutmix original. (**h**) Cutmix + free rotation. (**i**,**j**) Cutmix + monochrome transformation + free rotation.

Spatial augmentation was performed by applying free rotation to the eggs. Pixel augmentation was performed due to monochrome transformation (black and white), and the cutmix was made manually by mounting 100 images in the composition of images (Figure 6) from the other two thermal conditions described in Figure 1a,b. Note that YOLOv4 has the mosaic, and the cutmix augmentation techniques already included in the backbone and detector parts of the algorithm as a “bag of freebies”. Nevertheless, considering that we had only one object per image, when mounting similar objects that did not belong to our class of interest, we could have an honest model to detect difficult objects with more confidence.

#### 2.5.2. Training Parameters

The total training dataset composed of 3610 images was split into two groups in a proportion of 70:30; thus, 2527 images were used for training, and 1083 were used for validation.

**Figure 7:**
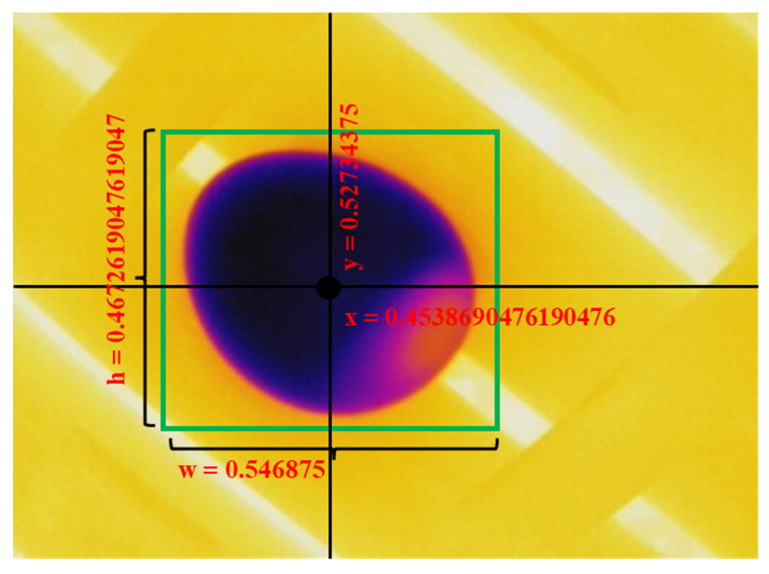
Image labeling for YOLO format. The coordinates x and y represent the position of the center point of the object in relation to the figure in pixels. H and W are the sizes of the bounding boxes in pixels.

To train YOLO object detection, the data were labeled according to YOLO format using a self-designed program that could give the bounding box and label coordinates x, y, height, and width (Figure 7). On the other hand, to train EfficientDet, the images were labeled using the open-source software LabelIMG, which gives bounding box coordinates in PASCAL VOC XML format.

To train the models, we used different frameworks. As mentioned earlier, YOLOv4 is embedded in the Darknet framework, while YOLOv5 is based on PyTorch, and EfficientDet is onboard TensorFlow.

YOLOv4 and EfficientDet were trained on a personal computer running Windows^®^ 10™ 64 bits, with an Intel^®^ Xeon™ E5-1607 processor, 32 GB of RAM, a NVIDIA^®^ GTX 1650™ 4 GB GPU, Python version 3.8.5, CUDA 10.1, cuDNN 7.6.5, OpenCV 4.4.0., TensorFlow 2.3.1, and TensorFlow-GPU 2.3.1. We trained YOLOv5 in the Google, LLC, Collab cloud environment with PyTorch 1.11.0 + cu102 and a 16 GB GPU Tesla T4.

Some hyperparameter values were different, such as the batch size and number of iterations (Table 2), as consequence of different frameworks. However, as our intention was to evaluate only the detection accuracy, training performance was not considered in this study.

#### 2.5.3. Evaluation Metrics

To validate the models and compare the results, we adopted the common metrics accepted and recognized by deep learning developers and the academy: the precision (P) is defined as the proportion of true positive (TP) detections in relation to false positive (FP) detections (Equation (2)), the recall (R) is TP in relation to false negative (FN) detections (Equation (3)), and the F1 score (Equation (4)) indicates the balance between precision and recall and is a good metric to compare the efficacy between models. The average precision (AP, Equation (5)) and mAP@0.5 (Equation (6)) are metrics adopted to evaluate the trained parameters of the models adopted by the PASCAL VOC challenge [58].

(2) $$P=\frac{\mathrm{TP}}{\mathrm{TP}+\mathrm{FP}}$$

(3) $$R=\frac{\mathrm{TP}}{\mathrm{TP}+\mathrm{FN}}$$

(4) $$F1\;\mathrm{score}=\frac{2\mathrm{PR}}{P+R}$$

(5) $$\mathrm{AP}=\frac{1}{11}\sum_{R_{i}}{\mathrm{PR}}_{i}$$

(6) $$\mathrm{mAP}=\frac{1}{N}\overset{N}{\underset{i=1}{\sum }}{\mathrm{AP}}_{i}$$

**Figure 8:**
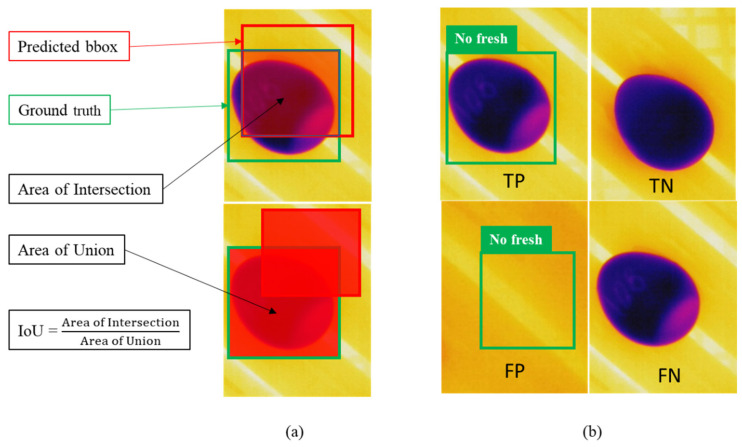
(**a**) IoU demonstrates the relation between the area of intersection and the area of union. (**b**) TP represents correct object detection, TN represents the correct object nondetection, FP is a false detection, and FN is a false negative, representing the case when the object should have been detected but was not.

It is important to note that concepts of true and false detections are determined according to the prediction bounding boxes (bbox) in relation to the reference label bbox, called the ground truth. The trueness is determined by setting the intersection over union (IoU), which calls for the proportion of a prediction in relation to the reference. Usually, the IoU is defined to be greater than 50% on the training setup (Figure 8).

## 3. Results

### 3.1. Correlation Test

**Figure 9:**
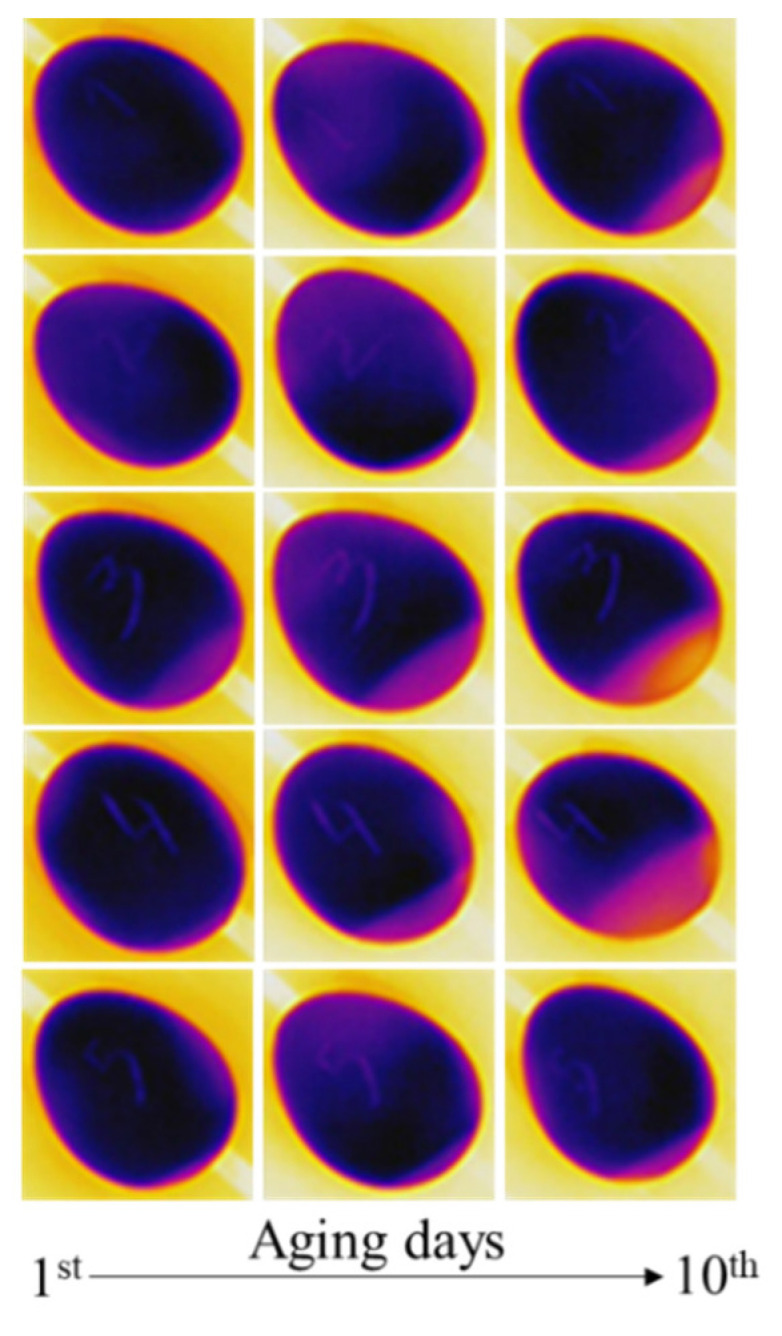
The aging effects on eggs after 10 days of storage under accelerated aging conditions of 28 °C and 60% humidity.

The thermal camera interprets the intensity of the infrared wavelength transmitted through the atmosphere. Cold eggs show different features according to the eggshell thickness and the conditions of the egg content, which may vary according to the storage conditions and their chemical properties. We observed that the air cell of eggs was visible when the egg temperature contrasted room temperature; some examples of thermal images from eggs are shown in Figure 9.

**Figure 10:**
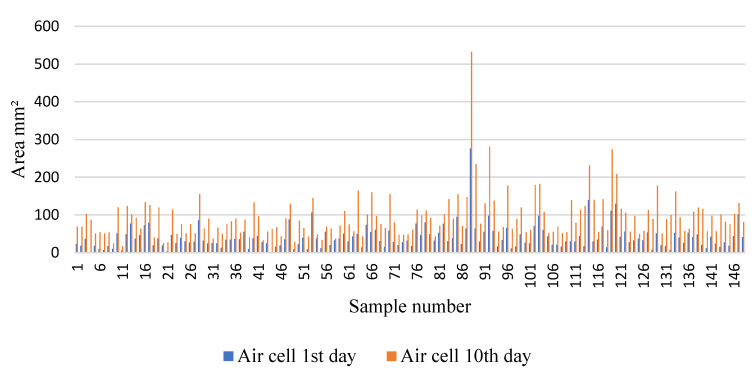
The variation in the air cell size for each egg between the first day of storage and the 10th day under accelerated degradation conditions.

The size of the air cell was measured according to the methodology described in Section 2.4. The results were tabulated in Microsoft^®^ Excel™ version 2209, and the statistics were calculated for the correlation test (Table 3). The variation in the air cell size occurred from the 1st day to the 10th day (Figure 10); all eggs were affected by aging with no exceptions, as we could observe the weight loss. However, some eggs were affected more than others, which is probably related to the composition of the eggshell that could provide resistance to the loss of water and gases.

The height was the real distance measured with a digital caliper, and the pixel length was the corresponding height distance in pixels provided by ImageJ software. We collected the pixel length for the 1st day and for the 10th day to ensure that any minimum modification on the position of eggs in relation to the camera did not interfere with the representative height in the images. The air cell area was determined by the program according to the reference length (pixel/mm) obtained from previous measurements.

The statistics represent that the difference in the weight [(weight 10th day) − (weight 1st day)] and the pixel area [(pixel area at 10th day) − (pixel area at 1st day)] was correlated by a Pearson’s test (Table 4).

The *p* value was very close to 0, which means we could reject the null hypothesis regarding the loss of weight which was not related to the enlargement of air cell size (Table 4). The alternative hypothesis was accepted in this case; in other words, a change in the weight could explain the variation in the air cell size, and the chance of that occurring by chance was close to zero.

**Figure 11:**
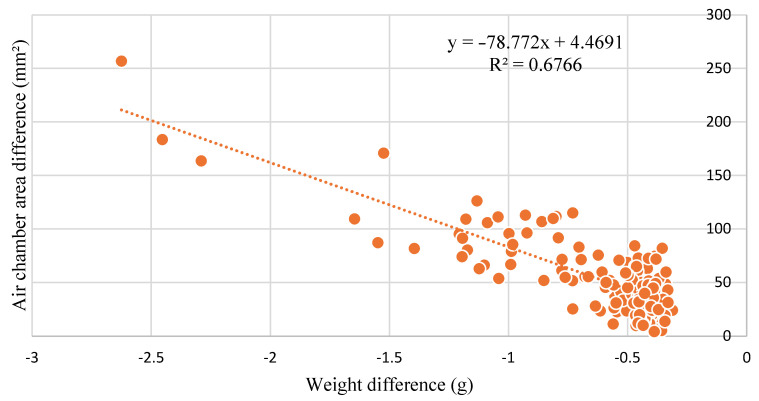
Correlation test for weight difference and air cell area.

The difference in weight and size was plotted in the graph (Figure 11). The average weight difference was 0.620 g over 10 days, which represents 6.26% of the average weight of fresh eggs. Quail eggs have different colors and pigmentation, making the detection of cracks and small fissures difficult to identify by human eyes. Some eggs that lost more weight than others may have been damaged at a location that was not identified before the experiment.

The graph shows that there was a negative correlation between the weight and size of air cells, with R^2^ = 0.6766. Hence, a negative correlation was observed, and an inverse relation between the two variables was found, which means that when the variable weight decreased, the air cell size increased.

**Figure 12:**
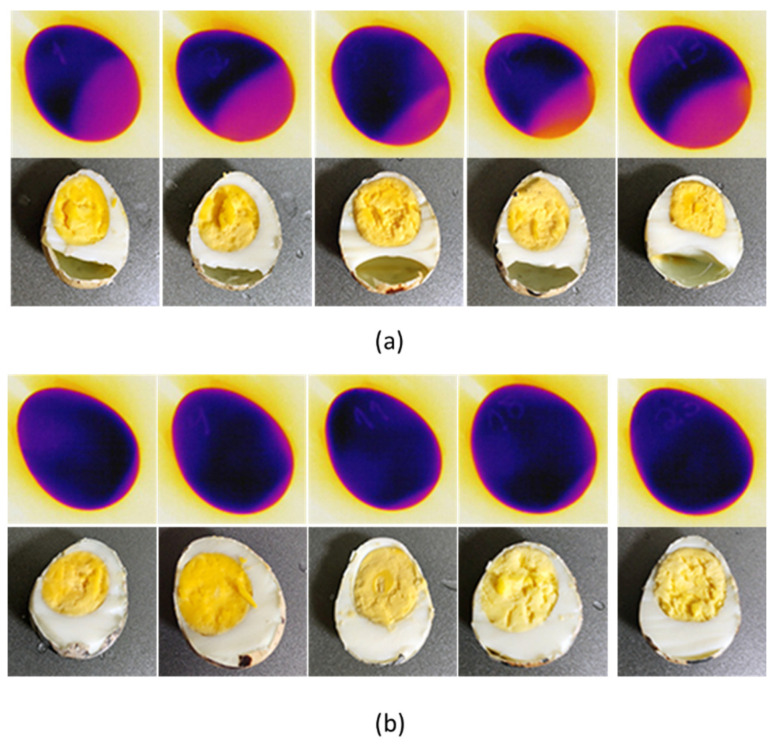
Egg assessment by boiling eggs and the corresponding thermal picture. (**a**) Nonfresh eggs present a large air cell. (**b**) Still fresh eggs show no chamber or reduced size compared to nonfresh eggs.

In addition to the statistical analysis, a visual assessment of the air cell features was provided from boiled eggs and corresponding thermal images. We noticed that the highlighted area on the thermal images corresponds to the cavity in the photo (Figure 12). The thermal images that did not show air cell features from the radiometric picture also did not show orifices in the boiled egg.

### 3.2. Computer Vision Model Prediction

#### 3.2.1. Training Results

**Figure 13:**
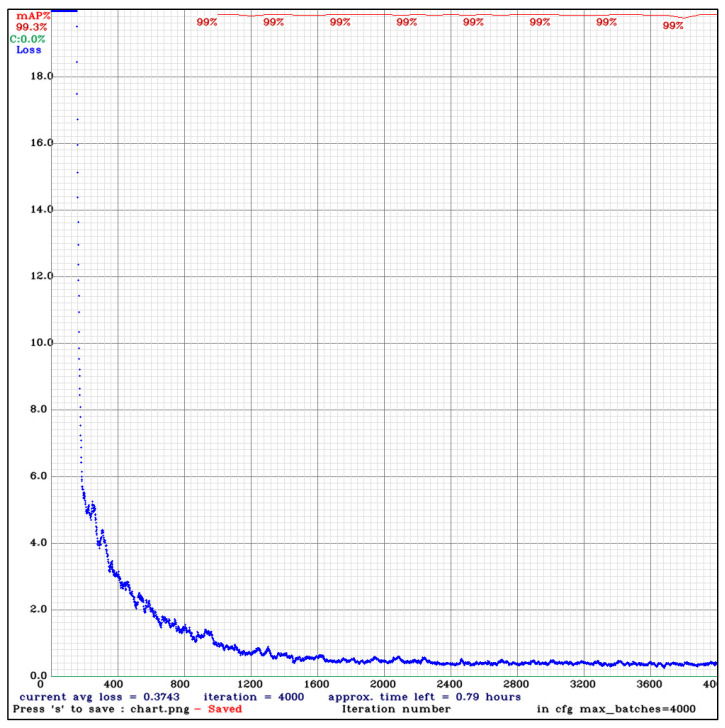
Training progress of the YOLOv4 object detection algorithm using the Darknet framework.

YOLOv4 object detection was trained for 4000 steps (Figure 13) in the Darknet framework based on the C programming language and CUDA. The training took 26 h to complete.

According to the graph, the average loss (which was the most important parameter to indicate the learning progress in deep learning) of the model reached the minimum average loss after 1600 steps (iteration batches), and the training could be interrupted. The mean average precision reached 99% but did not reach 100%, an indication of no overfitting of the model to the training dataset.

**Figure 14:**
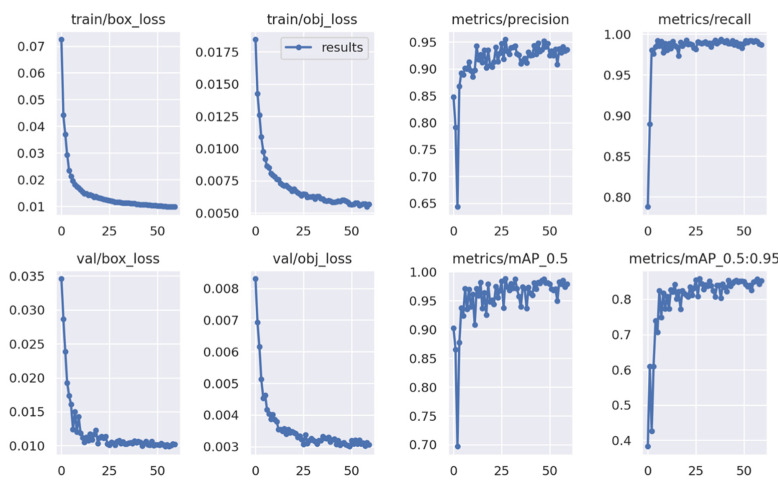
Training results from YOLOv5.

As mentioned in Section 2.5.2, YOLOv5 was trained in the PyTorch framework in the Google LLC Colab environment for approximately 1 h. The evaluation of PyTorch models is given in the Tensorboard™ application (Figure 14); such a tool was also used for the evaluation of EfficientDet. Different from the Darknet framework, the Tensorboard data report was more detailed and easier to understand.

YOLOv5 was trained for 60 epochs. However, when looking at the evaluation graphs, it was observed that after 25 epochs of training, the object loss reached a satisfactory value as a learning parameter, which was close to the minimum accuracy cost of the model as a supervised learning algorithm (Figure 14). In addition, when compared to the metric precision, the mAP could be considered stable after 25 epochs, with minimum improvements from visual analysis. Therefore, the model was trained more than enough epochs to reach the best results for the model. Similarly, the same case was observed for the YOLOv4 model.

Our third model, despite being part of the one-stage detector class, had a different architecture compared to the YOLO models. A notable difference was observed in the standard network size, which was higher (512 × 512), and was accomplished for the efficient detection of small objects for the purpose of scalable models.

**Figure 15:**
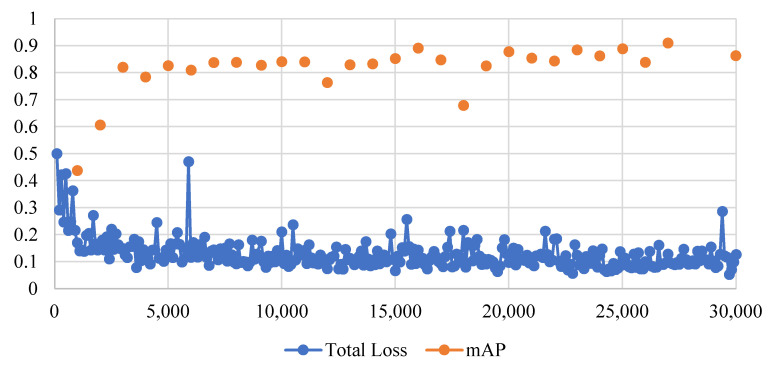
Training progress of EfficientDet during 30,000 steps and the mAP@0.50.

The training total loss (Figure 15) did not reduce considerably after 6000 steps, which means the training could be shorter than 30,000 steps, repeating the same results from the previous models (YOLOv4 and YOLOv5).

The precision and accuracy of each model (YOLOv4, YOLOv5, and EfficientDet) were not compromised by the training steps, and all models were trained for more time than was necessary to achieve mAP stability, which was the main parameter to evaluate the accuracy of the models (Table 5).

**Figure 16:**
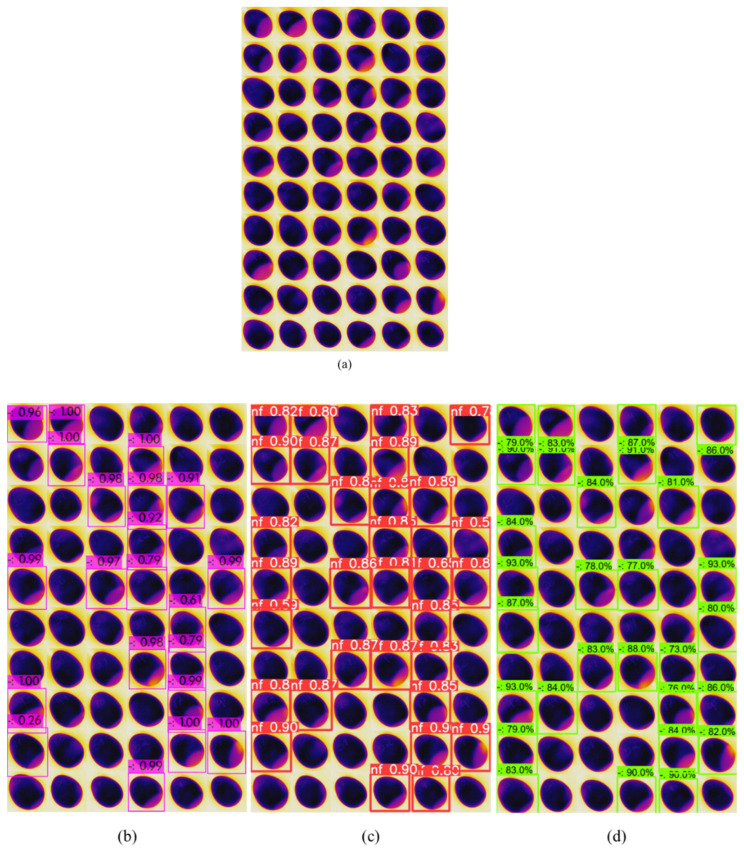
(**a**) The original images of 60 eggs were used to test the deep learning object detection models. (**b**–**d**) YOLOv4, YOLOv5, and EfficientDet models applied to the original testing dataset.

To test the models, the testing samples were grouped with all 60 quail eggs. The assessment of the results was performed by calculating the metrics of Equations (2)–(4) as given in Section 2.5.3. The accuracy assessment of the thermal images was made by boiling the eggs and cutting the longitudinal axis manually (Figure 12). The eggs with no or minimum air cells were considered fresh, the original image (Figure 16a) was used to test the YOLOv4, YOLOv5, and EfficientDet object detection models (Figure 16b–d).

The accuracy assessment of the eggs (longitudinal cutting of boiled eggs represented in the Figure 12) corresponded to the “correct answers” of a supervised learning algorithm and served as the main reference parameter for the calculation of the metrics’ precision, recall, and F1 score. In our dataset, 22 eggs were found as still fresh, which corresponded to the true negative (TN) detections (eggs that must not be detected as “nonfresh eggs”).

In Figure 16a, we referred to the original testing image where the eggs were kept in numerical order from 1 to 60 (from top left to bottom right) to test the object detection models’ accuracy on it. In Figure 16b–d, each bounding box (bbox) showed its detection score on the top right of the bbox under no threshold score for YOLOv4 and a 25% threshold for the YOLOv5 and EfficientDet models.

In the Figure 16b, we had the YOLOv4 detections (purple bbox) where we could observe one detection with 0.26 (26%) score, as an example of no threshold score. In the case of YOLOv4, it was important evidence to support the robustness and stability of the model when detecting only nonfresh eggs. The class name was omitted to make the visibility more effective, besides that, as we had only one class, the name was not relevant according to our purpose.

In Figure 16c, the results of testing YOLOv5 are presented; the class name “nf” stands for nonfresh class, as, for YOLOv5, it was a requirement input for training the model. Again, the score is shown on the top right position of each red bbox (minimum score was 0.5 or 50%).

EfficientDet testing results are shown in Figure 16d. The minimum confidence score was 0.73 (73%), that was the best result compared to other two models regarding the confidence score of the bbox. However, it was expected for the scalable model (designed to detect objects from low to high resolution images) and did not affect the final purpose of our investigation, which was based on the accuracy of nonfresh egg detection only.

The output predictions of the testing dataset (Table 6) were organized, and the metric calculations were computed.

The total bbox in Table 6 is the total number of detections for each model employed in this study; the true positive stands for the amount of bboxes that corresponds to nonfresh egg detections, in this case all bboxes for all models were correctly assigned to the class nonfresh; the true negatives were those eggs that must not be detected as they correspond to still fresh eggs. The false positive column calls for still fresh eggs wrongly classified as nonfresh eggs (no eggs were falsely classified for all three models); and, finally, the false negative column stands for the number of eggs that were supposed to be detected as nonfresh but were not. According to Table 5, we could calculate the precision, recall, and F1 score (Table 7).

**Figure 17:**
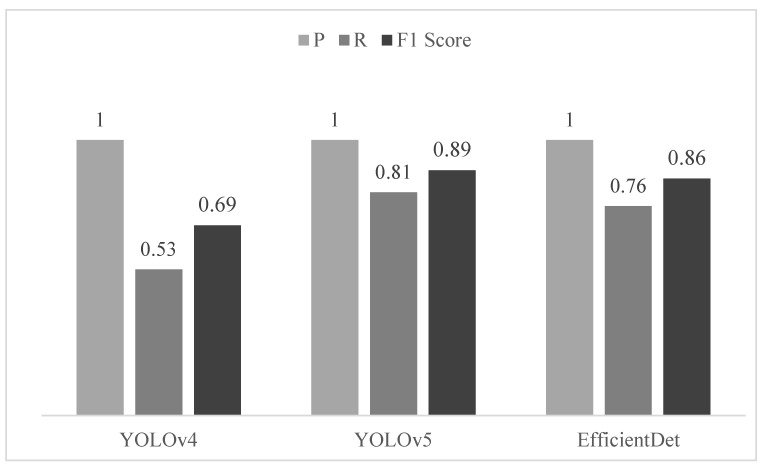
Results from the calculation of metrics to evaluate the testing dataset.

From the testing dataset, the precision metric (Equation (2)) for all three models was 1 or 100%, which means that all detections were correctly assigned for the class not fresh. The metric recall (Equation (3)) described the relation between the correct detections and undetected eggs that should be detected. The recall was higher for YOLOv5; in this case, we said that this model was responsible for detecting a greater quantity of nonfresh eggs properly (fewer false negative detections). For the F1 score (Equation (4)), this metric stood as the balance for precision and recall and could be understood as an equivalent metric for the mAP. As we did not label the testing dataset, the mAP could not be estimated in the frameworks; in this case, the F1 score was responsible for informing on which model performed better. YOLOv5 outperformed the other two (Figure 17), as well as the validation dataset.

#### 3.2.2. Revalidation of the Expiration Date

From the results of the DL prediction, we could calculate the revalidation proportion of the eggs 15 days after the labelled expiration date (Table 8). The revalidation was done considering the difference of total detections (total bbox) as “not fresh” (YOLOv4, YOLOv5, and EfficientDet) from the total amount of eggs (60). Therefore, the deep learning (DL) revalidation was given by {[(TN+FN)/Total eggs] × 100}, and the true revalidation was defined as [(TN/Total eggs) × 100]. The revalidation error was determined by [(DL revalidation − True Revalidation)].

## 4. Discussion

In this work, we used a thermal camera and proposed a new methodology to detect the freshness of eggs according to the air cell size. Thermal cameras have the ability to interpret the intensity of infrared wavelengths transmitted through the atmosphere. Hence, considering that CO_2_ is heavier than atmospheric air (at the same temperature and pressure), when contrasting distinct temperatures of cold eggs and warm room temperature, the CO_2_ and the composition of other gases in the air cell produce a spectral signature transmitted from the eggs that can be detected by the radiometric sensors of the thermal cameras. We called this method the “thermal imaging contrast technique”. When using this method, the identification of the air cell was easy, fast, and approachable to identify stale and not fresh eggs. Therefore, this method can be used in real time for high-throughput applications at the industrial level, especially when combined with deep learning object detection algorithms in automated systems, as demonstrated in this work.

During this study, the pH of the albumen or the yolk were not measured during the storage period. However, the literature shows that for hen eggs, the pH of the albumen may increase slightly more than the yolk pH [59], and this modification can be reduced under controlled atmospheric systems by injecting CO_2_ into the storage room [60]. Additional factors can also contribute to potentializing the chemical transformations of the albumen and yolk, such as genotype, quail feed composition, diseases, age of parental flock, and environmental conditions.

Methods able to perform real time and nondestructive analysis can contribute to the development of quail industry farming and the post harvesting process to keep the quality and safety for the consumers. In this study, it was noticed that some eggs from the same groups were less fresh than others. From this observation, we can presume that eggs collected from the same quail may vary in eggshell composition, such as thickness and hardness.

In the correlation test, we observed that the standard deviation of real measurements and the pixel measurements were very different in magnitude because the pixel measurements were relative to the manual line tracing on marked points, which means that the line traced between two points may change the length of pixels when connecting the line to the top and bottom points of the eggs. Nevertheless, we traced a line three times and used the mean to define our pixel length; consequently, the error was reduced for our measurements.

For the second part of our study, by using deep learning object detection, the prediction of not fresh quail eggs with high accuracy was possible. When comparing the three models, the best results were obtained from YOLOv5, followed by EfficientDet and YOLOv4. Deep learning-based models for image recognition and localization are being upgraded continuously as the demand for applications for this tool increases in many sectors of our daily lives.

The comparison between deep learning algorithms has demonstrated that improvements on object detection algorithms can reduce the error of reclassification of nonfresh eggs. However, as thermal cameras have low resolution, further improvements on thermal sensing can also enhance the efficiency and accuracy of deep learning-based computer vision systems; therefore, the tradeoff between equipment and algorithms should be considered as a drawback.

While training the deep learning models, some parameters can influence the speed and accuracy of the model, and the most important parameter is the network size. The network size of YOLOv4 and YOLOv5 was set to 416 × 416 pixels because these models use the same architecture; however, the batch size was different due to the dependence on hardware resources, especially processing power (GPU). For EfficientDet, the minimum network size was 512 × 512 due to the scalable feature architecture, and the batch size was reliable for TensorFlow™ processing. As our hardware did not allow training YOLOv4 with a network size larger than 416 × 416, only the accuracy between models was compared, thus, the speed deployment was not taken into consideration in this study.

## 5. Conclusions

The air cell is one of the most important parameters to qualify the freshness of eggs. The thermal camera was able to interpret the infrared wavelength intensities transmitted through the atmosphere from the eggshell pores and then, by the contrast technique, highlight the portion where the gases were accumulated on the large base of the eggs as a consequence of the storage period. As the aging process affects the size of the air cell, the lack of freshness was correlated with the air cell size (R^2^ = 0.676). The combination of thermal camera imagery and deep learning object detection algorithms could identify nonfresh quail eggs with high accuracy; besides that, our investigation has demonstrated their potential to compose automatic systems for freshness assessment at industry and civil levels. We tested our model on the eggs after the expiration date, the YOLOv4, YOLOv5, and EfficientDet models could detect nonfresh eggs with an F1 score of 0.69, 0.89, and 0.86, respectively. The best model (YOLOv5) demonstrated that 48.33% of eggs could have their labelled date extended at least 15 days, with an error rate of 11.67%. EfficientDet and YOLOv4 showed reclassification proportions of 51.67% and 66.67% and an error rate of 15% and 30%, respectively.

The developed methodology was reported as a fast and nondestructive way to assess the freshness of quail eggs according to the detection of air cell size; the methodology itself can be extended for industrial applications, supermarkets, and restaurants to relabel eggs for extended consumption periods and minimize the postharvest poultry production losses.

The main limitation of the methodology developed is regarding how long the expiry date could be extended for. In this regard, further studies should be addressed including the assessment of eggshell thickness for prediction of ideal shelf-life of quail eggs and other avian eggs as well.

## Figures and Tables

**Table 1:** YOLOv4, YOLOv5, and EfficientDet deep learning object detection algorithms basic architecture comparison.

Model	Backbone	Neck	Head	Loss Function	Training Framework
YOLOv4	CSPDarknet53	PANet SPP block	YOLO layer	Binary cross entropy	Darknet
YOLOv5	CSPDarknet53	PANet Modified FPN	YOLO layer	Binary cross entropy and logits function	PyTorch
EfficientDet	EfficientNet	BiFPN	Box Prediction net	Focal loss	TensorFlow

**Table 2:** Training parameters for each object detection algorithm used in this work.

Algorithm	Batch Size	Input Size	Momentum	Number of Iterations
YOLOv4	64	416 × 416	0.949	4000 batches
YOLOv5	16	416 × 416	0.937	60 epochs
EfficientDet	4	512 × 512	0.899	30,000 steps

**Table 3:** Data exploration analysis for weight and air cell size of stored eggs.

Metric	Height (mm)	1st Day Pixel Length	10th Day Pixel Length	Chamber Area 1st Day	Chamber Area 10th Day	Weight (g) 1st Day	Weight (g) 10th Day
Average	30.993	147.678	141.122	40.464	93.763	9.902	9.282
SD	1.306	16.963	18.897	32.071	59.454	0.930	0.968

**Table 4:** Statistical analysis for the Pearson correlation between the weight and air cell size.

Weight Difference (Mean)	Pixel Area Difference (Mean)	n Observations	Degrees of Freedom (n-2)	T-Statistic	Coefficient (r)	*p* Value
−0.620	53.300	148	146	17.47713	−0.82256	1.3 × 10^−37^

**Table 5:** Evaluation of object detection models for IoU 0.5.

Model	Precision (P)	Recall (R)	F1 Score	mAP@0.50
YOLOv4	0.99	0.99	0.99	99.24%
YOLOv5	0.99	0.99	0.99	99.5%
EfficientDet	0.95	0.73	0.82	95.0%

**Table 6:** Testing dataset metrics for accuracy performance evaluation.

Model	Total bbox	True Positive (TP)	True Negative (TN)	False-Positive (FP)	False Negative (FN)
YOLOv4	20	20	22	0	18
YOLOv5	31	31	22	0	7
EfficientDet	29	29	22	0	9

**Table 7:** Testing dataset accuracy evaluation with no threshold for YOLOv4 and threshold 0.5 for YOLOv5 and EfficientDet.

Model	Precision (P)	Recall (R)	F1 Score
YOLOv4	1	0.53	0.69
YOLOv5	1	0.81	0.89
EfficientDet	1	0.76	0.86

**Table 8:** Revalidation amount according to DL object detection algorithms.

Model	Total Eggs	Not Fresh (bbox)	DL Fresh (TN + FN)	True Negative (TN)	DL Revalidation	True Revalidation	Revalidation Error
YOLOv4	60	20	40	22	66.67%	36.67%	30.00%
YOLOv5	60	31	29	22	48.33%	36.67%	11.67%
EfficientDet	60	29	31	22	51.67%	36.67%	15.00%

## Data Availability

The dataset that was generated and analyzed during this study is available from the corresponding author upon reasonable request, but restrictions apply to the data reproducibility and commercially confident details.
